# Supplementary material for: Genome-wide rare variant score associates with morphological subtypes of autism spectrum disorder
Source: Nat Commun. 2022 Oct 29;13:6463. doi: 10.1038/s41467-022-34112-z (PMC9617891; doi:10.1038/s41467-022-34112-z)
Supplement: Supplementary file 3 — Description of Additional Supplementary Files [file 41467_2022_34112_MOESM3_ESM.pdf]

Description of Additional Supplementary Files

File Name: Supplementary Data 1
Description: Discovery Cohort Characteristics

File Name: Supplementary Data 2
Description: Summary of WGS quality and number of variants detected

File Name: Supplementary Data 3
Description: Validation status of de novo exonic SNVs and indels in discovery cohort

File Name: Supplementary Data 4
Description: Validation status of de novo exonic CNVs in discovery cohort

File Name: Supplementary Data 5
Description: Clinically significant and ASD candidate SNVs and indels in discovery cohort

File Name: Supplementary Data 6
Description: Clinically significant and ASD candidate CNVs, SVs, and tandem repeat expansions in discovery cohort

File Name: Supplementary Data 7
Description: Phenotype description of 69 probands with clinically significant or ASD-candidate variants in discovery cohort

File Name: Supplementary Data 8
Description: Prevalence of ASD variants in morphologic subtypes of ASD in the discovery cohort

File Name: Supplementary Data 9
Description: Prevalance of SNVs, indels and CNVs in 43 genesets among ASD subtypes in the discovery cohort

File Name: Supplementary Data 10
Description: Prevalance of SNVs, indels and CNVs in noncoding regions among ASD subtypes in the discovery cohort

File Name: Supplementary Data 11
Description: : Coefficients of significant gene sets and regions in the discovery cohort (classified by gold standard dysmorphology) using 10x30-fold cross validation

File Name: Supplementary Data 12
Description: Phenotype, GRVS and PRS information for discovery cohort

File Name: Supplementary Data 13
Description: Average scores for clinically significant variants, all and remaining ASD-relevant variants in the discovery cohort that occur in ASD relevant regions identified by gene set enrichment

File Name: Supplementary Data 14
Description: Coefficients of significant gene sets and regions in the ADM-classified discovery cohort under 10x30-fold cross validation*

File Name: Supplementary Data 15
Description: Coefficients of gene sets and regions with P< 0.1 in the ADMclassified discovery cohort

File Name: Supplementary Data 16
Description: Phenotype, GRVS and PRS information for replication cohort

File Name: Supplementary Data 17
Description: Clinically significant and ASD candidate SNVs and indels in the replication cohort

File Name: Supplementary Data 18
Description: Clinically significant and ASD candidate CNVs in replication cohort

File Name: Supplementary Data 19
Description: Variant predictors and databases used for variant annotation

File Name: Supplementary Data 20
Description: noncoding regions examined in this study

File Name: Supplementary Data 21
Description: Gene sets used in gene set enrichment analysis
