# Supplementary material for: Genome-wide rare variant score associates with morphological subtypes of autism spectrum disorder
Source: Nat Commun. 2022 Oct 29;13:6463. doi: 10.1038/s41467-022-34112-z (PMC9617891; doi:10.1038/s41467-022-34112-z)
Supplement: Supplementary file 5 — Reporting Summary [file 41467_2022_34112_MOESM5_ESM.pdf]

nature portfolio

Corresponding author(s): Stephen Scherer
Bridget Fernandez

Last updated by author(s): Oct 3, 2022

Reporting Summary

Nature Portfolio wishes to improve the reproducibility of the work that we publish. This form provides structure for consistency and transparency in reporting. For further information on Nature Portfolio policies, see our Editorial Policies and the Editorial Policy Checklist.

Statistics

For all statistical analyses, confirm that the following items are present in the figure legend, table legend, main text, or Methods section.

n/a	Confirmed	
☐	☒	The exact sample size (*n*) for each experimental group/condition, given as a discrete number and unit of measurement
☒	☐	A statement on whether measurements were taken from distinct samples or whether the same sample was measured repeatedly
☐	☒	The statistical test(s) used AND whether they are one- or two-sided<br>*Only common tests should be described solely by name; describe more complex techniques in the Methods section.*
☐	☒	A description of all covariates tested
☐	☒	A description of any assumptions or corrections, such as tests of normality and adjustment for multiple comparisons
☐	☒	A full description of the statistical parameters including central tendency (e.g. means) or other basic estimates (e.g. regression coefficient) AND variation (e.g. standard deviation) or associated estimates of uncertainty (e.g. confidence intervals)
☐	☒	For null hypothesis testing, the test statistic (e.g. *F*, *t*, *r*) with confidence intervals, effect sizes, degrees of freedom and *P* value noted<br>*Give P values as exact values whenever suitable.*
☒	☐	For Bayesian analysis, information on the choice of priors and Markov chain Monte Carlo settings
☒	☐	For hierarchical and complex designs, identification of the appropriate level for tests and full reporting of outcomes
☐	☒	Estimates of effect sizes (e.g. Cohen's *d*, Pearson's *r*), indicating how they were calculated

Our web collection on statistics for biologists contains articles on many of the points above.

Software and code

Policy information about availability of computer code

Data collection	No software was used for data collection
Data analysis	R v3.5.1 (statistical analysis)<br>R scripts used in this study are available at https://github.com/naibank/GRVS_ASD<br>PLINK v1.9.0b6.4 (common variant analysis)<br>PRSice-2 v2.2.0 (common variant analysis)<br>KING v2.2.5 (relatedness analysis)<br>bcftool v1.6, EIGENSOFT v6.0beta and ADMIXTURE v1.3 (ancestry analysis)<br>BWA 0.7.12 (alignment)<br>ExpansionHunter Denovo v0.7.0 (detection of tandem repeats)<br>GATK Haplotype Caller41 v3.4.6 (detection of SNVs and smaller indels)<br>denovogear 1.1.1 (detection of de novo SNVs and indels)<br>ERDS 1.1 and CNVnator 0.3.2 (detection of CNVs)<br>Manta v0.29.6 (detection of structural variants)<br>ANNOVAR Feb 2016 version (variant annotation)

For manuscripts utilizing custom algorithms or software that are central to the research but not yet described in published literature, software must be made available to editors and reviewers. We strongly encourage code deposition in a community repository (e.g. GitHub). See the Nature Portfolio guidelines for submitting code & software for further information.

Data

Policy information about availability of data

All manuscripts must include a data availability statement. This statement should provide the following information, where applicable:

- Accession codes, unique identifiers, or web links for publicly available datasets
- A description of any restrictions on data availability
- For clinical datasets or third party data, please ensure that the statement adheres to our policy

The WGS data generated in this study have been deposited into controlled access research databases, as further described below, because this is the type of data sharing that was approved by the study participants. Access to FASTQ data for samples in the discovery cohort that were consented for MSSNG can be obtained by completing the data access agreement: https://research.mss.ng. Access to FASTQ data for samples in the discovery cohort not consented for MSSNG, as well as VCF files for sequence-level variants for all samples in the discovery cohort are available at European Genome-Phenome Archive (accession EGAS00001005753: https://ega-archive.org/studies/EGAS00001005753). Access to data for the replication cohort can be obtained by completing data access agreement (https://www.sfari.org/resource/sfari-base), as was done for this study. The clinical data generated in this study are provided in the Supplementary Data 7,12, and 16. Public databases used in this study can be accessed using the following links: 1000 Genomes Project (https://www.internationalgenome.org/), NHLBI Exome Sequencing Project (https://evs.gs.washington.edu/EVS/), gnomAD (https://gnomad.broadinstitute.org/), Human Phenotype Ontology (https://hpo.jax.org/app/), Mouse Phenotype Ontology (http://www.informatics.jax.org/vocab/mp_ontology), ClinVar (https://www.ncbi.nlm.nih.gov/clinvar/), Human Gene Mutation Database (https://www.hgmd.cf.ac.uk/), Clinical Genomics Database (https://research.nhgri.nih.gov/CGD/), and Online Mendelian Inheritance in Man (https://www.omim.org/).

Human research participants

Policy information about studies involving human research participants and Sex and Gender in Research.

Reporting on sex and gender	We worked to ensure sex balance in the recruitment of human research participants (the male:female ratio for individuals with ASD in this study closely mirrors the well-established 4:1 sex bias in ASD).
Population characteristics	All population demographics of subjects including ethnicity and sex are provided in the supplementary material and is available from the respective databases.
Recruitment	The discovery cohort consists of children residing in the Canadian province of Newfoundland and Labrador, recruited from one of three developmental team assessment clinics between 2010 and 2018. Participation was offered whether or not the child had syndromic features or a known genetic diagnosis.
Ethics oversight	The study was approved by Newfoundland's Health Research Ethics Boards (HREB# 2003.027) and SickKids Research Ethics Board (REB#0019980189).

Note that full information on the approval of the study protocol must also be provided in the manuscript.

Field-specific reporting

Please select the one below that is the best fit for your research. If you are not sure, read the appropriate sections before making your selection.

☒ Life sciences ☐ Behavioural & social sciences ☐ Ecological, evolutionary & environmental sciences

For a reference copy of the document with all sections, see nature.com/documents/nr-reporting-summary-flat.pdf

Life sciences study design

All studies must disclose on these points even when the disclosure is negative.

Sample size	We categorized 325 Canadian children with ASD into dysmorphic and nondysmorphic subgroups and used whole-genome sequences (WGS) and detailed clinical morphology data to: 1) develop a genome-wide rare variant score (GRVS) to measure the relationship between rare variants and morphology, and 2) examine the contribution of rare and common variants in morphological ASD subtypes. We did not perform a formal sample size calculation but using a cohort of 259 ASD probands, we had previously demonstrated a significant difference in ASD-associated rare variants between dysmorphic and nondysmorphic probands; the variants were identified using a combination of exome sequencing and microarray (Tammimies K et al, JAMA, 2015). The discovery cohort used in current manuscript was larger in size (325 unrelated probands from families that had been consented for genome sequencing). Based on our previous work, we anticipated that 60% of the cohort would be classified as nondysmorphic and 40% as dysmorphic, and that the dysmorphic group would be significantly enriched for rare variants, which would allow us to move forward with developing a genome-wide rare variant score.
Data exclusions	Data was not excluded from the study.
Replication	Findings were replicated using WGS data from 442 ASD probands with accompanying morphology data from the Simons Simplex Collection
Randomization	Not applicable. This is an observational study. There are no exposures or interventions that require randomization.

Blinding	Not applicable. This is an observational study. There are no exposures or interventions that require blinding.

Reporting for specific materials, systems and methods

We require information from authors about some types of materials, experimental systems and methods used in many studies. Here, indicate whether each material, system or method listed is relevant to your study. If you are not sure if a list item applies to your research, read the appropriate section before selecting a response.

Materials & experimental systems

n/a	Involved in the study
☒	☐ Antibodies
☐	☒ Eukaryotic cell lines
☒	☐ Palaeontology and archaeology
☒	☐ Animals and other organisms
☐	☒ Clinical data
☒	☐ Dual use research of concern

Methods

n/a	Involved in the study
☒	☐ ChIP-seq
☒	☐ Flow cytometry
☒	☐ MRI-based neuroimaging

Eukaryotic cell lines

Policy information about cell lines and Sex and Gender in Research

Cell line source(s)	DNA was obtained from lymphoblast-derived cell lines from a total of 15 individuals (6 probands and 9 parents). Four individuals are female and 11 are male.
Authentication	None of the cell lines used were authenticated.
Mycoplasma contamination	Cell lines were not tested for mycoplasma contamination.
Commonly misidentified lines (See ICLAC register)	N/A

Clinical data

Policy information about clinical studies

All manuscripts should comply with the ICMJE guidelines for publication of clinical research and a completed CONSORT checklist must be included with all submissions.

Clinical trial registration	N/A
Study protocol	N/A
Data collection	N/A
Outcomes	N/A
