# Supplementary material for: Genome-wide rare variant score associates with morphological subtypes of autism spectrum disorder
Source: Nat Commun. 2022 Oct 29;13:6463. doi: 10.1038/s41467-022-34112-z (PMC9617891; doi:10.1038/s41467-022-34112-z)
Supplement: Supplementary file 6 — Source Data [file 41467_2022_34112_MOESM6_ESM.zip › Source data/Supplementary Figures 8-9.docx]

**Original gel for Supplementary Figure 8**

Family 3-0439 Chr16 Inversion/insertion

1 2 3 4 5 6 7 8 9 10 11 12


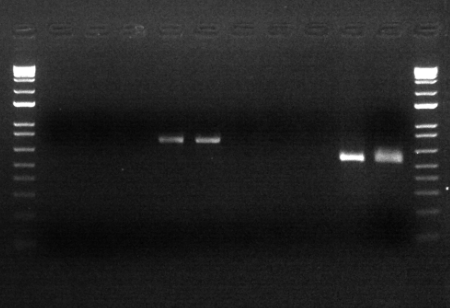


500 bp

850 bp

Lane 1= 1 kb+ ladder Lane 7= -ve control PHF21A-R/PHF21A-RC-F

Lane 2 = -ve control PHF21A-F/RC-Rev Lane 8= Control DNA 10851

Lane 3= control DNA 10851 lane 9= 3-0439-100-172704

Lane 4=3-0439-100-172704 Lane 10= 3-0439-101-172705

Lane 5= 3-0439-101-172705 Lane 11=3-0439-000-172703

Lane 6= 3-0439-000-172703 Lane 12= 1 kb+ ladder

Expected size= 741 bp expected size= 483 bp

**Original gels for Supplementary Figure 9**

1 2 3 4 5 6 7


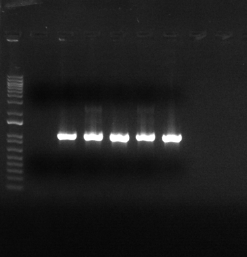


Lane1= 1 kb+ ladder

Lane2= -ve control FGFR2-F-R

Lane3= control 10851

Lane4= 3-0728-100 263192

Lane5 = 3-0728-101 263191

Lane 6= 3-0728-000 263188

Lane 7= 3-0728-001 265597

PCR expected size = 1.014kb for reference product on FGFR2

PCR approx. expected size for alt allele= ~2383

1 2 3 4 5


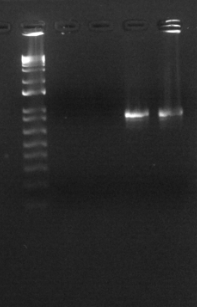


1 kb

850 bp

Lane1= 1 kb+ ladder

Lane2= -ve control

Lane3= 10851 ref control

Lane4= 3-0728-001 Mother

Lane5= 3-0728-000 Proband

Nested PCR

Expected size =approx. 900bp

1 2 3 4 5 6 7 8


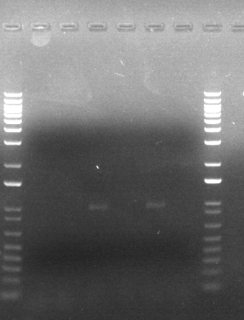


Lane 1= 1 kb+ ladder

Lane 2= -ve control Pseudo NPMI-F/FGF2-R

Lane 3= control 10851

Lane 4=3-0728-100-263192

Lane 5= 3-0728-101-263191

Lane 6= 3-0728-000-263188

Lane 7= 3-0728-001-265597

Lane 8= 1 kb+ ladder

Expected size = 975 bp

Nested PCR

1 kb

1 2 3 4 5 6 7 8 9 10 11 12 13 14 15 16 17


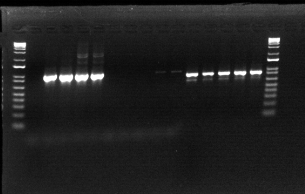


2 kb

1 kb

850 bp

Lane 1= 1kb+ ladder lane 7= -ve control PseudoF-FGF2-R

Lane 2= -ve control FGFR2-F-R Lane 8= control 10851

Lane 3= control 10851 Lane 9= 3-0209-100 93339

Lane 4= 3-0209-100 93339 Lane 10= 3-0209-101 93340

Lane 5= 3-0209-101 93340 Lane 11= 3-0209-000 241173W

Lane 6= 3-0209-000 241173W expect 975 bp , No Amplification in control

Expect 1 kb product and possibly 2.3 kb product

Lane 12= -ve control Sect A- Pseudo RC-Rev (contamination redo pcr)

Lane 13= control 10851

Lane 14= 3-0209-100 93339

Lane 15= 3-0209-101 93340

Lane 16=3-0209-000 241173W

Expect 900 bp product

Note gel box runs slanted to the left. All bands are the same size


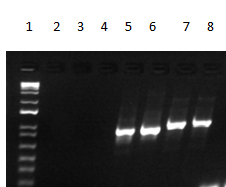


Lane 1= 1kb+ ladder

Lane 2= -ve control

Lane 3= 10851 control

Lane 4= 3-0209-100 93339 Mother

Lane 5= 3-0209-101 93340 Father

Lane 6= 3-0209-000 241173W proband

Lane 7 = 3-0209-101 93340 father using FGFr2-F-R fragment as template

Lane 8 = 3-0209-000 241173W proband using FGFr2-F-R fragment as template
